# Editorial: New analytical strategies in plant metabolites analysis

**DOI:** 10.3389/fpls.2023.1332622

**Published:** 2023-11-21

**Authors:** Jianing Mi, Xiongjie Zheng

**Affiliations:** ^1^ State Key Laboratory of Traditional Chinese Medicine Syndrome, The Second Affiliated Hospital of Guangzhou University of Chinese Medicine, Guangzhou, China; ^2^ The BioActives Lab, Center for Desert Agriculture, King Abdullah University of Science and Technology, Thuwal, Saudi Arabia

**Keywords:** plant metabolites analysis, new analytical strategies, spatiotemporal metabolomics, mass spectrometry, cryo-TOF-SIMS, weighted gene co-expression network analysis, multivariate statistical analysis

Over hundreds of millions of years, plants have undergone robust evolutionary adaptations from their algae ancestors, developing various mechanisms to cope with diverse types of stress. It has become evident that plants produce a wide array of primary and secondary metabolites, which serve as chemical signals for communication with their environment for survival. These metabolites have significant physiological and ecological functions. However, the analysis of plant metabolites poses several challenges: 1) the detection of metabolites that exhibit an extensive range, vast diversity, relatively low abundance, and dynamic nature; 2) comprehensive data analysis and interpretation.

The utilization of advanced analytical techniques, such as mass spectrometry (MS), plays a crucial role in enabling the accurate and reliable detection and quantification of metabolites. For instance, a study conducted by Yan et al. highlights the application of ultra-high-performance liquid chromatography-high-resolution tandem mass spectrometry (UHPLC-HRMS/MS) and UHPLC coupled to triple quadrupole MS (UHPLC-QQQ-MS) techniques in investigating the dynamic accumulation of glucosinolates and their derivatives in radish (*Raphanus sativus*) at various growth stages. This analytical approach allowed for comprehensive insights into the biosynthetic pathways of radish-specific glucosinolates. By leveraging the resolution and sensitivity of the integration of UHPLC-HRMS/MS and UHPLC-QQQ-MS techniques, researchers were able to gain a deeper understanding of the biosynthesis and accumulation patterns of these specific metabolites in radish plants. Furthermore, the release of specific metabolites by plant root systems influences the microenvironment of the rhizosphere, subsequently impacting plant root metabolism and exudate secretion. Heuermann et al. conducted a comprehensive profiling of primary and secondary metabolites in root exudates collected from field-grown and hydroponically cultured plants using an LC-MS-based approach. Interestingly, the study highlighted that the metabolites released in root exudates were specific to the cultivation conditions, emphasizing the critical role of the cultivation environment in influencing root exudate secretion. Moreover, environmental factors can significantly contribute to the regional variability of secondary metabolites in medicinal plants. For example, Chen et al. employed UPLC-MS and chemometrics analysis to study metabolites from *Anisodus tanguticus* (Maxim.) Pascher originating from different geographical locations, revealing geographical traceability.

Furthermore, the complex and intricate nature of metabolomics data necessitates the application of advanced statistical and bioinformatics tools to extract valuable insights from the vast amount of generated data, uncovering patterns, correlations, and meaningful connections that help elucidate the biological context underlying the metabolomic profiles. Integration of metabolite analysis with statistical and bioinformatics methods is commonly employed by researchers to uncover the beneficial pharmacological functions of plant secondary metabolites in humans. For instance, Chen et al.‘s work integrated metabolomics and network pharmacology to elucidate the “multicomponent-multitarget” mechanism of *A. tanguticus*. Similarly, Ning et al. utilized widely targeted metabolomics and weighted gene co-expression network analysis to identify characteristic metabolites of different hemp seed varieties, highlighting the antioxidant activities primarily attributed to flavonoids and phenolics.

Plant metabolism is a dynamic process, and metabolite distribution varies across different plant tissues, exhibiting spatiotemporal variation and tissue specificity. Consequently, these fluctuations in metabolic profiles ultimately shape plant phenotypes. However, the exploration of spatiotemporal variations in plant metabolites has remained a challenge. Researchers have faced difficulties due to the complex matrix of plant samples, harsh sample preparation requirements, and limitations of available instruments, all of which hinder the identification and structural characterization of plant metabolites at a spatial level. However, in the study by He et al., time-of-flight secondary ion mass spectrometry (TOF-SIMS) image analysis technology was employed for the first time to investigate the distribution of metabolites in the cross-section of Coptis rhizome. This study successfully imaged and quantified the spatial distribution and content of nine active compounds, revealing an increase in these compounds with the growth years of the rhizome. The advantage of non-destructive sample detection offered by the TOF-SIMS image method makes it an effective tool for understanding the spatial distribution of active compounds on the surface of traditional Chinese medicine. In addition, Gong et al. developed a cryo-TOF-SIMS and scanning electron microscopy (cryo-TOF-SIMS/SEM) strategy to elucidate the microscopic distribution of eight alkaloids on the transverse surface of freeze-fixed *Phellodendron amurense* Rupr. stems during the fall and summer. Supported by HPLC analysis of these alkaloids, the study revealed that the relative contents of the eight alkaloids varied in different positions with changing seasons, suggesting their potential roles in the physiological processes of the plant or its response to environmental conditions. Gong et al.‘s study shed light on the spatiotemporal variations of *P. amurense* alkaloids, providing a crucial foundation for further research on the genes or enzymes involved in biosynthetic pathways and the specific functions of alkaloids in plants.

In summary, this Research Topic provides valuable insights into the latest analytical strategies, which significantly enhance our ability to understand the dynamic spatiotemporal profiles of plant metabolites. Moreover, our Research Topic offers valuable perspectives on statistical analysis and data visualization in metabolite analysis.

## Author contributions

JM: Project administration, Writing – original draft, Writing – review & editing. XZ: Project administration, Writing – review & editing.

